# Redox Chemistry and [Au(CN)_2_^-^] in the Formation of Gold Metabolites

**DOI:** 10.1155/MBD.1994.351

**Published:** 1994

**Authors:** G. Frank Shaw III, Sabine Schraa, Ernst Gleichmann, Yash Paul Grover, Lothar Dunemann, Annapurna Jagarlamudi

**Affiliations:** 1 Medizinisches Institut für Umwelthygiene Department of Immunobiology Postfach 10 37 51 Düsseldorf D-40225 Germany; 2 The Department of Chemistry The University of Wisconsin-Milwaukee PO. Box 413 Milwaukee WI53201-0413 USA

## Abstract

The role of hypochlorite ion, which can be generated by the enzyme myleoperoxidase, in the biochemistry of gold(I) anti-arthritic drugs was investigated.  Sodium hypochlorite (OCl^−^) directly and rapidly oxidizes AuSTm, Au(CN)_2_^-^, AuSTg (gold thioglucose) and auranofin (Et_3_PAuSATg). The resulting gold(III) species were detected by an Ion Chromotography Ion-Pairing technique that was developed to distinguish gold(I) and gold(III). Formation of Au(III) was also demonstrated spectrophotometrically after the conversion to AuCl_4_^−^. The reactions of AuSTm, AuSTg, and auranofin are complex and gold(III) appears only after the initial oxidation of the thiolate (and phosphine) ligands.

The enzymatic reaction, using MPO with H_2_O_2_ and Cl^−^ as substrates, leads to slow oxidation of Au(CN)_2_^-^, AuSTm or AuSTg. The extent and rate of reaction depend on the concentrations of MPO, H_2_O_2_, and Au(I). The continued presence of Au(I) during the initial stages of reaction (oxidation of the thiolates in AuSTm and AuSTg) and the conversion to Au(III) in the latter stages of the reaction were demonstrated.

Au(CN)_2_^-^, a gold metabolite, binds tightly to serum albumin. Unlike other gold(I) complexes, aurocyanide reacts almost negligibly at Cys-34 *via* ligand exchange. Instead, there is a strong association (K_1_ = 5.5 × 10^4^ and K_2_ = 7.0 × 10^3^; n_1_ = 0.8 and n_2_ = 3) of intact Au(CN)_2_^-^. The full extent of binding is revealed only by equilibrium methods such as NMR or ultrafiltration; the bound gold dissociates extensively on conventional gel-exclusion columns and partially on Penefesky spun columns.

The immunological and pharmacological significance of these results are discussed.


### REDOX CHEMISTRY AND [$Au(CN)_2^-$] IN THE FORMATION OF GOLD METABOLITES

G. Frank Shaw III, Sabine Schraa, Ernst Gleichmann, Yash Paul Grover, Lothar Dunemann, and Annapurna Jagarlamudi

Medizinisches Institut für Umwelthygiene, Department of Immunobiology, Postfach 10 37 51, D-40225 Düsseldorf, Germany and
The Department of Chemistry, The University of Wisconsin-Milwaukee, PO Box 413, Milwaukee, WI53201-0413, USA

The role of hypochlorite ion, which can be generated by the enzyme myleoperoxidase, in the biochemistry of gold(I) anti-arthritic drugs was investigated. Sodium hypochlorite ($OCl^-$) directly and rapidly oxidizes AuSTm, $Au(CN)_2^-$, AuSTg (gold thioglucose) and auranofin ($Et_3PAuSATg$). The resulting gold(III) species were detected by an Ion Chromotography Ion-Pairing technique that was developed to distinguish gold(I) and gold(III). Formation of Au(III) was also demonstrated spectrophotometrically after the conversion to $AuCl_4^-$. The reactions of AuSTm, AuSTg, and auranofin are complex and gold(III) appears only after the initial oxidation of the thiolate (and phosphine) ligands.

The enzymatic reaction, using MPO with $H_2O_2$ and $Cl^-$ as substrates, leads to slow oxidation of $Au(CN)_2^-$, AuSTm or AuSTg. The extent and rate of reaction depend on the concentrations of MPO, $H_2O_2$, and Au(I). The continued presence of Au(I) during the initial stages of reaction (oxidation of the thiolates in AuSTm and AuSTg) and the conversion to Au(III) in the latter stages of the reaction were demonstrated.

$Au(CN)_2^-$, a gold metabolite, binds tightly to serum albumin. Unlike other gold(I) complexes, aurocyanide reacts almost negligibly at Cys-34 *via* ligand exchange. Instead, there is a strong association ($K_1 = 5.5 \times 10^4$ and $K_2 = 7.0 \times 10^3$; $n_1 = 0.8$ and $n_2 = 3$) of intact $Au(CN)_2^-$. The full extent of binding is revealed only by equilibrium methods such as NMR or ultrafiltration; the bound gold dissociates extensively on conventional gel-exclusion columns and partially on Penefesky spun columns.

The immunological and pharmacological significance of these results are discussed.

### INTRODUCTION

The mechanism of action of anti-arthritic gold drugs has been a matter of speculation and research for almost six decades. Strong evidence suggests that the bulk of gold *in vivo*, especially the circulating metabolites, remain gold(I), the oxidation state present in myochrysin, solganol, and auranofin [1]. Thus, it is interesting that immunological results in animal models [2] and humans [3] suggest a role for gold(III). This prompted us to reexamine the cursory report [4] that gold(I) can be oxidized by hypochlorite ion generated from $H_2O_2$ and $Cl^-$ in the presence of the enzyme myleoperoxidase (MPO) which is produced and released by phagocytic cells.

The oxidation of gold(I) thiomalate (AuSTm) to gold(III) by myleoperoxidase-generated hypochlorite ion was briefly described in an abstract by Beverly and Couri [4]. Their reactions were effected under conditions mimicking the oxidative burst of phagocytic immune cells. Although further details were reported in a thesis [5], no full report has appeared in the standard literature and these studies were limited to the single gold complex, AuSTm. The protein chemistry of gold complexes has been explored in some detail [1] but relatively less is known about its biological redox chemistry. A number of lines of evidence suggest that gold(III) is readily reduced to gold(I)

under conditions prevailing *in vivo* [6,7,8] and that bulk gold in tissues is gold(I) even after administration of gold(III) to laboratory animals [8]. Nonetheless, many reviews point out the feasibility of oxidation [9-13] and possible reactions have been reported [4,14,15]. Thus, the observation that gold(III), but not gold(I), induces secondary immune responses in laboratory animals [2] and human patients [3] treated with gold(I) drugs suggests that gold(III) is indeed generated *in vivo*.

Since generation of gold(III) by stimulated macrophages or polymorphoneutrophils has the potential to dramatically alter the metabolism and anti-arthritic activity, as well as the immunogenicity, of gold drugs, we have investigated the direct reaction of sodium hypochlorite with gold thiomalate, gold(I)thioglucose, aurocyanide and auranofin. The MPO-mediated oxidation of these species was also examined. To facilitate the analysis, we developed an Ion Chromotography Ion-Pairing technique to quantitate the +I and +III oxidation states of gold after forming dicyanogold(I) and tetracyanogold(III), respectively, *in situ*.

Conditions under which the same enzyme, MPO, can produce cyanide from thiocyanate and subsequently generate aurocyanide [$Au(CN)_2^-$] from AuSTm have also been reported [16]. Therefore, it was also of interest to examine the reaction(s) of aurocyanide with serum albumin, the principle gold binding protein in blood.

### EXPERIMENTAL PROCEDURES

**Materials.** Gold Thiomalate ($H_2$AuSTm; aurothioapfelsäure, lot #192129) was a kind gift from E. Tosse GmbH & Co., Hamburg. Sodium hypochlorite (5% solution, Sigma, St. Louis) was diluted daily to 0.6-5 mM in dd $H_2O$ or PBS and quanititated using the taurine/thionitrobenzoate method [17]. Thio-nitrobenzoate (TNB) solutions (3-4 mM) were prepared by $NaBH_4$ reduction of 5,5'-dithiobis-(2-nitrobenzoic acid) (DTNB), stored frozen, and quantitated daily by their absorbance at 412 nm $\epsilon_{412}$ = 13,600 $M^{-1}$ $cm^{-1}$). $KAu(CN)_2$ was a gift of Dr. Anne Arendt (nee Hormann) [18]. Reactions were carried out in phosphate buffered saline solution (PBS = 50 mM phosphate, 132 mM NaCl, pH 7.4). HCl (1.0 M standardized) was from E. Merck (Darmstadt Germany). Solutions (10 mM) of KCN (Sigma, Germany ) were prepared fresh daily.

### Chemical Oxidation

Reactions of OCl- with AuSTm, $Au(STm)_2^-$, AuSTg, $Au(CN)_2^-$ or auranofin were carried out in PBS, pH 7.4 at room temperature. Concentrations were typically 10-80 $\mu$M AuSTm and 10-900 $\mu$M $OCl^-$. Gold(III) was analyzed as $AuCl_4^-$ after acidification of reaction mixtures to pH 1 with 1M HCl ($\epsilon_{316}$ = 4860 $M^{-1}$ $cm^{-1}$). Neither AuSTm nor $OCl^-$ absorbs significantly in this wavelength region at acid pH. Gold(I) could be detected qualitatively by conversion to $Au(CN)_2^-$ in a solution containing 1 mM KCN. Intense bands at 204 and 211 nm ($\epsilon_{204}$ = 12,850 and $\epsilon_{211}$ = 10,230 $M^{-1}$ $cm^{-1}$) and weaker bands at 230 and 240 nm ($\epsilon_{230}$ = 3663 and $\epsilon_{240}$ = 3441 $M^{-1}$ $cm^{-1}$) provide a distinctive signature. Since the formation constants for $Au(CN)_2^-$ are unusually large, displacement of simple monodentate ligands is complete [19-21].

### Enzyme Mediated Oxidation

Solutions containing typically 10 $\mu$g/mL MPO were reacted with gold complexes ([$Au(CN)_2^-$], AuSTm, AuSTg or auranofin; 10-40 $\mu$M) in PBS, pH 7.4, with $H_2O_2$ (20-100 $\mu$M). The $H_2O_2$ was the last reagent added to initiate the reaction. Temperature was 25°C except as noted otherwise. The reactions were monitored by the absorbance changes at 220 nm. In some cases, the reaction mixtures were analyzed spectrophotometrically for Au(III) after addition of HCl, or by IC after addition of 1mM KCN to the reaction mixture. Either reagent at the concentrations used terminates the enzymatic reaction.

### Ion Chromatography

A Dionex 20001/SP module equipped with a Chromjet integrator, Ion-Pac Guard Pre-column (P/N 39567), Ion-Pac MPIC-NS1 column (P/N 35321), Micromembrane Suppressor (P/N 37106), and Conductivity Cell Detector was employed. The eluant was 50/50 V/V $CH_3CN/H_2O$ (Grade 1

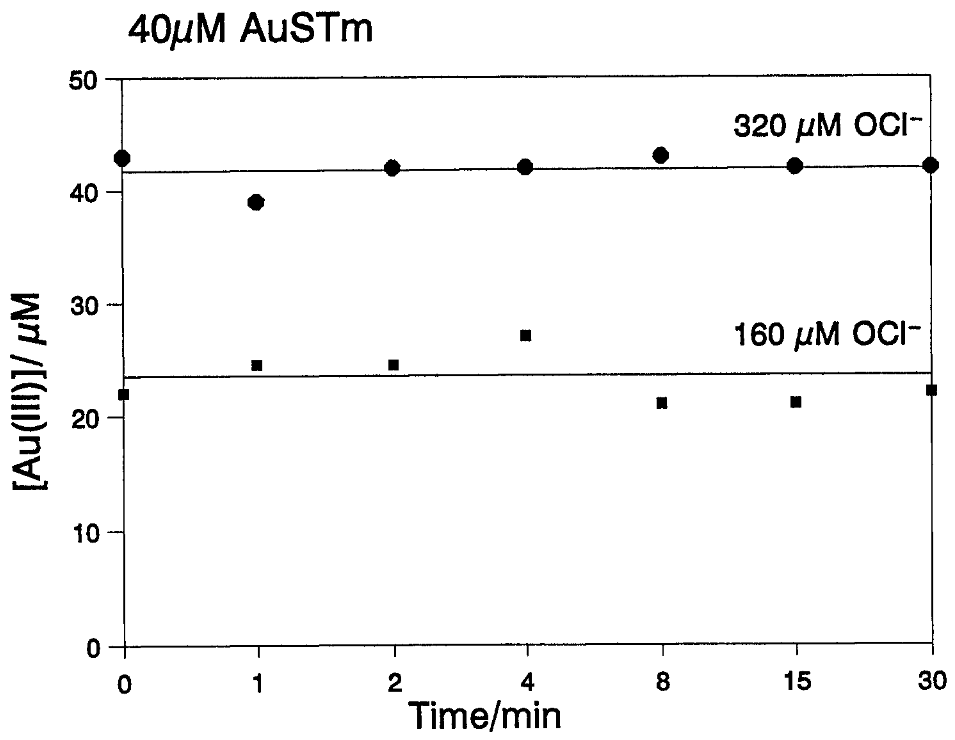

**Figure 1.** Time Independence of Au(III) formation from AuSTm (40 μM) and 4 or 8 equivalents of $OCl^-$. Conditions: $[OCl^-]$ = 160 or 320 μM; Phosphate buffered saline solution (PBS), pH 7.4, 25°C.

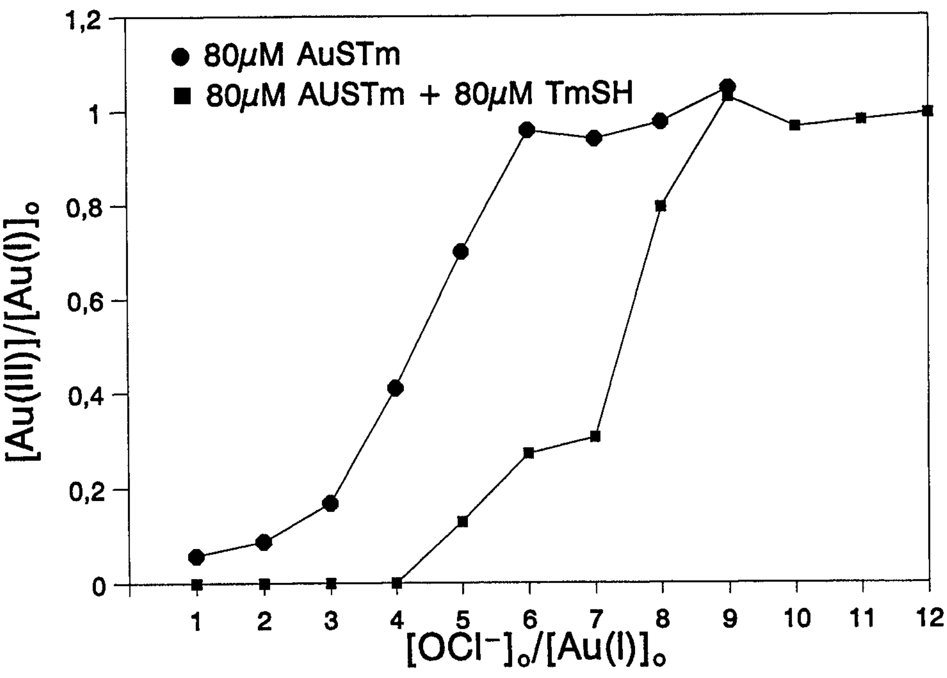

**Figure 2.** Redox Titration of AuSTm and $Au(STm)_2^-$ by $OCl^-$. Reaction mixtures were acidified to 0.10 M HCl one minute after mixing and analyzed spectrophotometrically for Au(III) by the absorbance at 316 nm. Conditions: [Au(I)] = 80 μM; $[OCl^-]$ = 80-800 μM; PBS, pH 7.4, 25°C.

deionized) with 1 mM tetrabutylammonium hydroxide, 500 $\mu$M $Na_2CO_3$. Samples were introduced *via* a 50 $\mu$L loop and eluted at 1 mL/min. $Au(CN)_2^-$ and $Au(CN)_4^-$ standard solutions were prepared in water and used for elution time and sensitivity calibrations.

### RESULTS

### Oxidation AuSTm by Hypochlorite

Reactions of $OCl^-$ and AuSTm were initially carried out with 40 $\mu$M gold and 174 $\mu$M $OCl^-$. Repetitive scans at one minute intervals demonstrated the rapid conversion of AuSTm into a new species with a more intense absorbance in the 220 nm region followed by a slower change in the new absorption band. Because the spectrum of gold(III) in aqueous solution at neutral pH results from a mixture of hydroxo and aquo species in a complex equilibrium [21], the reaction mixture was subsequently acidified with HCl to a concentration of 100 mM. The resulting spectrum revealed a band at 316 nm characteristic of $AuCl_4^-$ which demonstrates oxidation of the gold(I) to gold(III). The absorbance corresponded to 22.2 $\mu$M Au(III) (40% conversion) in the original solution after 40 min. No gold(III) was detected in control solutions containing 160 or 320 $\mu$M $OCl^-$ but no AuSTm, or 40 $\mu$M AuSTm but no $OCl^-$.

The incomplete reaction observed above could be due to slow kinetics of oxidation, an equilibrium process, or competing oxidation of the thiomalate ligand. The first possibility was eliminated by studying extent of reaction between 40 $\mu$M AuSTm and 160 or 320 $\mu$M $OCl^-$ over 30 minutes. As shown in Figure 1 the extent of reaction is invariant over the time span 1 to 30 minutes,

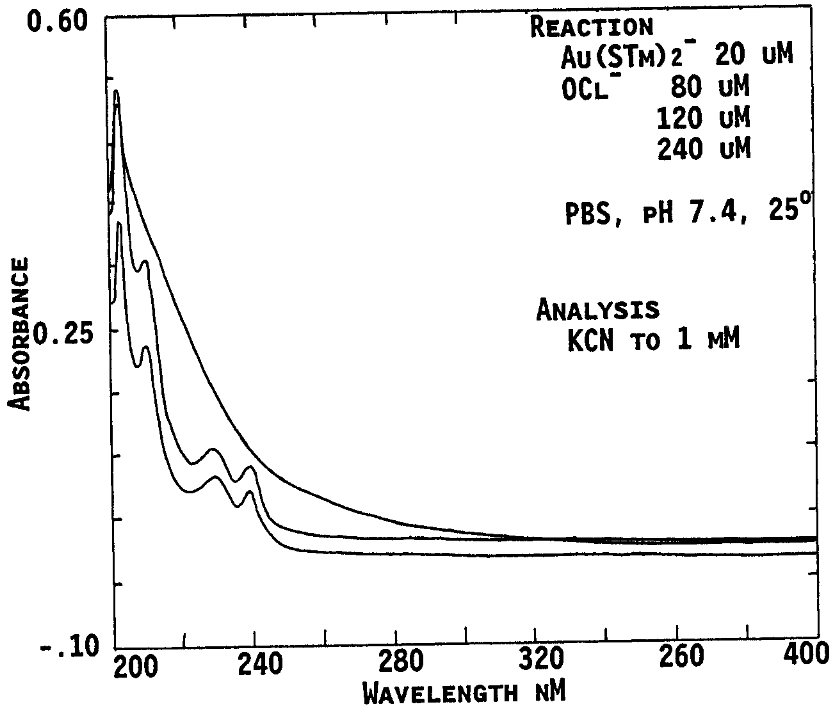

**Figure 3.** Spectrophotometric demonstration of unoxidized gold(I) as $Au(CN)_2^-$. $Au(STm)_2^-$ (20 $\mu$M) was treated with 80, 120 or 240 $\mu$M $OCl^-$ and then adjusted to 1 mM KCN by adding 0.11 vol of a 10 mM KCN stock solution. Sharp bands between 204 and 240 nM characteristic of $Au(CN)_2^-$ are observed in the solutions with 4 or 6 equivalents of $OCl^-$ but not after adding 12 equiv. Conditions: PBS, pH 7.4, 25°C.

but dependent on the concentration of $OCl^-$. 320 μM hypochlorite (8 $OCl^-$/Au(I)) drove the reaction to completion, but 160 μM $OCl^-$ (4 $OCl^-$/Au(I)) yielded only 55% conversion.

Next a titration was performed measuring the production of Au(III) as a function of the $OCl^-$ concentration. As shown in Figure 2, the reaction consumes between 2.5 and 3 equivalents of hypochlorite before the onset of Au oxidation. This corresponds to the oxidation of the thiomalate to its sulfonic acid form, $TmSO_3^-$, before the gold can be oxidized:

$$AuSTm + 3OCl^- \longrightarrow AuCl_2^- + TmSO_3^- + Cl^-$$

$$AuCl_2^- + OCl^- + 3H^+ \longrightarrow Au^{III} + H_2O + 3Cl^-$$

The ability of the thiomalate to neutralize three equivalents of hypochlorite was confirmed by an analogous tritration of bis(thiomalato)gold(I), generated in situ from reaction of one equivalent of thiomalate with AuSTm. In this case extensive formation of gold(III) occurred only after five equivalents of hypochlorite were added, Fig. 2.

The presence of unoxidized gold(I) when fewer than 3 or 6 equivalents of $OCl^-$ had been added to AuSTm or $Au(STm)_2^-$, respectively, was confirmed by addition of cyanide ion (to 1 mM final concentration). Au(I) is converted to $Au(CN)_2^-$, which has four unusually sharp and strong diagnostic bands in the region 200-240 nm, Fig. 3. The formation constant of $Au(CN)_2^-$ is unusually large [23,24]. Facile and complete displacement of thiolates from Au(I) by cyanide has been previously demonstrated [19-21]. Thus, the presence of Au(I) can be demonstrated qualitatively in the early but not the later stages of the titration.

**Ion Chromatographic Analysis of Au(I) & Au(III)**

To eliminate the ambiguity inherent in UV-visible assessment of the presence of gold(I) and gold(III), a more reliable and quantitative method was developed. Ion chromotography [25] using an anion column with ion pairing technique was found to be suitable for the detection of gold(I) and gold(III) as their respective cyanide complexes:

$$Au^{I}(CN)_2^- \quad \text{and} \quad Au^{III}(CN)_4^-$$

Conductivity measurements provide a sensitive and reproducible method to quantitate the two ions as they elute from the anion column. $Au(CN)_2^-$ eluted as a sharp peak at about 3.9 min and $Au(CN)_2^-$ as a broader peak at about 6.7 min. The limits of detection were 55 and 110 nM, respectively; and the linear ranges were found to be 3 to 45 μM and 0.5 to 6 μM. Table I gives the concentrations of gold(I) and gold(III) in solutions of AuSTm after reaction with 1 to 3 equivalents of $OCl^-$. The IC data clearly confirm that the formation of gold(I) commences only after extensive oxidation of the thiomalate ligand.

**Table I. Ion Chromatographic Analysis of AuSTm Oxidation by $OCl^-$**

| Reactants[a] | | | Final Concentrations (μg/mL)[b] | |
|---|---|---|---|---|
| [AuSTm]/μM | [$OCl^-$]/μM | $OCl^-$/Au | [Au(I)] | [Au(III)] |
| 80 | 80 | 1 | 0.59 | 0.00 |
| 80 | 160 | 2 | 0.51 | 0.00 |
| 80 | 240 | 3 | 0.42 | 0.42 |

[a]Reactions were run in PBS at 25μC. [b]Analysis for gold after adding KCN to a final $CN^-$ concentration of 1 mM and further diluting to the linear range of the technique.

**Hypochlorite Oxidation of Other Gold(I) Compounds**

AuSTg is also oxidized to Au(III) by $OCl^-$ in a reaction that shares the characteristics of the AuSTm reaction: preliminary oxidation of the thiolate ligand and then conversion to gold(III). The reaction may be slightly slower as the reaction reaches its endpoint after 2 minutes, not within the first minute as for AuSTm.

The hypochlorite oxidation of $Au(CN)_2^-$ was clearly demonstrated by the loss of the characteristic UV bands at 204, 211, 230 and 240 nm [30]. These results indicate that the reactions of AuSTm and AuSTg are not limited to the gold(I) thiolates but are a characteristic of gold(I) complexes in general. The presence of Au(III) was confirmed by the absorbance of $AuCl_4^-$ and by IC.

Auranofin ($Et_3PAuSAtg$), which contains a triethylphosphine ligand as well as a thiolate (2,3,4,6-tetra-O-acetyl-1-thiolato-ß-D-glucose) was also examined. The formation of Au(III) commenced in this case after four equivalents of hypochlorite were added, which is consistent with the oxidation of the phosphine to $Et_3PO$ and thiolate to sulfonate preceeding the oxidation of gold(I) to gold(III). $^{31}P$ NMR results confirm the formation of $Et_3PO$ (A.A. Isab, A. Jagarlamudi, and C.F. Shaw III, unpublished result).

**Myleoperoxidase Mediated Oxidation of Gold**

Myleoperoxidase is an important enzyme of the oxidative burst. It is present in neutrophils and to a lesser extent other phagocytic immune cells. During the oxidative burst, it utilizes hydrogen peroxide to generate hypochlorite from chloride ion:

$$H_2O_2 + Cl^- \longrightarrow OCl^- + H_2O.$$

It is likely then that hypochlorite generated by myleoperoxidase can then effect the oxidation of gold(I) to gold(III) under *in vivo* conditions. To explore this possibility, gold(I) compounds were exposed to MPO in the presence of its substrates, $H_2O_2$ and chloride ion, to ascertain the feasibility and the extent of oxidation that would occur. PBS was used as the reaction medium because the degranulation of neutrophils releases MPO into the extracellular space which has a neutral pH.

The reaction of AuSTm with $H_2O_2$ and $Cl^-$ in the presence of MPO led to a complex pattern of changes in the absorbance monitored at 220 nm, Fig. 4. There was an initial decrease over about 1-2 minutes followed by an increase over the next 2-3 minutes after which the absorbance leveled off or declined very slowly. The same pattern was observed by Beverly [5]. Control reactions in which $Cl^-$, $H_2O_2$, AuSTm or MPO were omitted from the mixture failed to produce the characteristic decrease and increase in absorbance. Beverly [5] had hypothesized that the initial decrease is due to the initial oxidation of the thiomalate which disrupts the gold-thiolate bonds giving rise to broad absorbances in the region 200-240 nm. The inorganic studies above and IC data below provide strong support for this interpretation. The subsequent increase in absorbance at 220 nm is due to the formation of Au(III) as a mixed chloro, aquo, hydroxo complex. Increasing the AuSTm concentration increased the time required to achieve the minima and maxima in absorbance, further indicating that both phases involve this complex.

The presence of Au(III) in the product mixture was confirmed by UV-vis spectroscopy after conversion to $AuCl_4^-$ by adding HCl to 0.1 M and by Ion Chromatography after conversion to $Au(CN)_4^-$ by adding HCN to 1 mM. Fig. 5 shows ion chromatographs of the same reactant mixture examined in Fig. 4 before and at 1, 2, 3 and 10 min. following the initiation of reaction. The decrease in the gold(I) signal (eluting 3.9 min after injection) and the increase in the gold(III) signal (6.7 min after injection) are clearly delineated. The final sample taken 10 minutes after initiating the reaction shows no detectable gold(I), indicating that complete oxidation was effected.

Oxidation of $Au(CN)_2^-$ and AuSTg have also been examined in the presence of MPO, $H_2O_2$ and $Cl^-$ to generate $OCl^-$. The aurocyanide reaction proceeds with an absorbance increase at 220 nm after a brief lag phase (ca. 15 sec). The increase is completed within about 2 minutes and shows a gradual decrease of the next 5-10 minutes. The conversion to Au(III) was confirmed by demonstrating the presence $AuCl_4^-$ after adding HCl to 0.10 M. As the AuSTg reaction proceeds, the 220 nM absorbance increases and then levels off after about 2-3 min. Thus, it

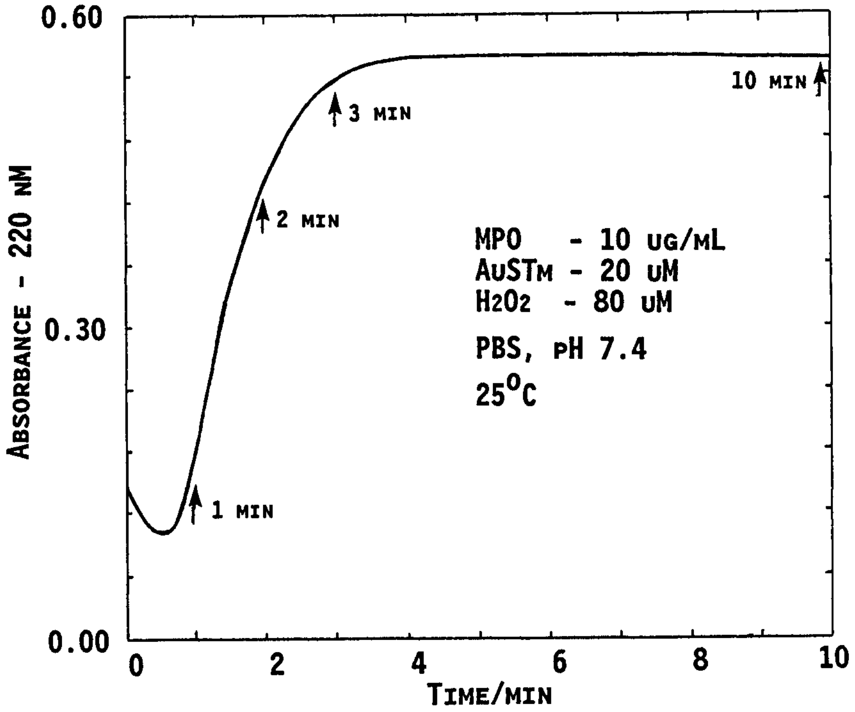

**Figure 4.** Oxidation of AuSTm by $OCl^-$ generated by MPO from $Cl^-$ and $H_2O_2$. The reaction was conducted at 25°C in PBS, pH 7.4 and monitored at 220 nm; [MPO] = 10 $\mu$g/mL; [$Cl^-$] = 132 mM; [AuSTm] = 20 $\mu$M; [$H_2O_2$] = 80 $\mu$M. Reactions were initiated by addition of $H_2O_2$. In some reactions, half the mixture was withheld from the spectrometer and sampled before and during (0, 1, 2, 3 & 10 min) the reaction for IC analysis (Fig. 5).

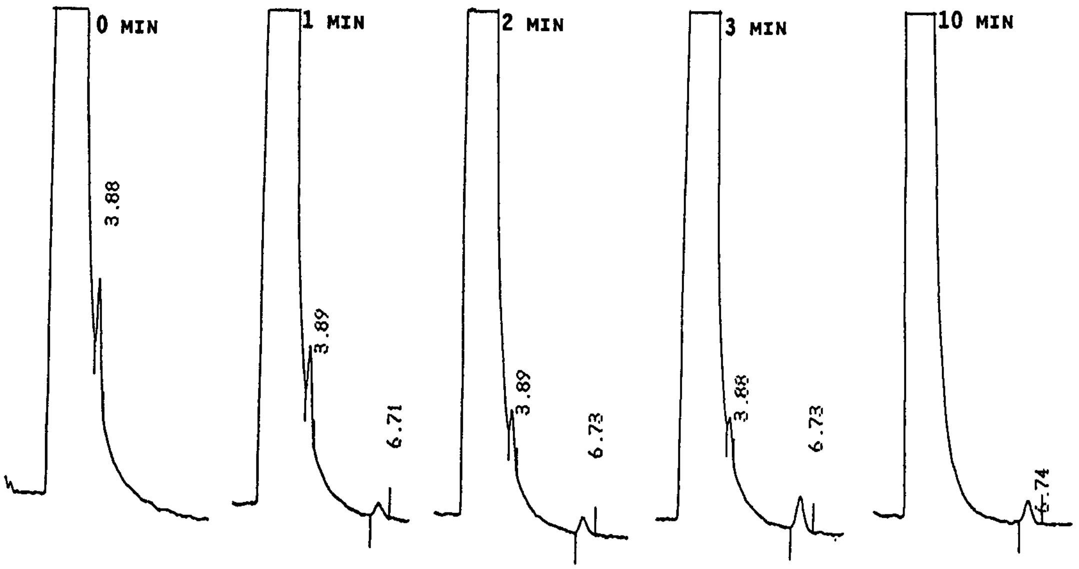

**Figure 5.** Ion Chromatographic analysis of the changing gold(I) and gold(III) content during an MPO-mediated oxidation of AuSTm (as in Fig 4.). Aliquots removed before and during the reaction (0, 1, 2, 3, 10 min) were immediately adjusted to 1 mM with KCN to quench the enzymatic reaction and to convert all Au(I) and Au(III) species present to $Au(CN)_2^-$ and $Au(CN)_4^-$, respectively. Before analysis they were diluted to the linear range of the technique.

resembles the increasing portion of the trace in Fig 4. Au(III) was again confirmed spectrophotometrcally.

**Binding of Aurocyanide to Serum Albumin**

$Au(CN)_2^-$ can be generated in the presence of $SCN^-$ under conditions approximating those found *in vivo* during chrysotherapy [23] and is found in patients who are non-smokers [24]. These recent reports demonstrate that $Au(CN)_2^-$ is an important metabolite and that its role in gold therapy warrants investigation. Many gold complexes react with serum albumin at cys-34 to form thermodynamically robust complexes which are transported in the blood [1]. $Au(CN)_2^-$, unlike AuSTm or AuSTg, accummulates rapidly and extensively in red blood cells, especially in patients who smoke [26]. Thus, it is important and necessary to characterize the interaction(s) of aurocyanide and serum albumin in order to understand the transport and distribution of this metabolite.

Preliminary studies [Dr. A. A. Isab, 27] with $Au(^{13}CN)_2^-$ by C-13 NMR spectroscopy suggested extensive association with albumin that was independent of Cys-34, while chromatographic studies with $Au(^{14}CN)_2^-$ suggested a very limited extent of reaction *via* ligand exchange at Cys-34 to to form AlbSAuCN. To resolve the inherent contradictions additional studies were undertaken using two additional separation methods. The first is Penefsky chromatography which rapidly separates free and protein-bound metal ions over a gel-exclusion column using centrifugal force [28]. The second method is membrane ultrafiltration which preserves the equilibrium between bound and free metal ion during the separation procedure [29].

**Table II. Binding of [$Au(CN)_2^-$] to Bovine Serum Albumin Determined By 3 Independent Separation Methods.**

| | Au/BSA after separation | | |
|---|---|---|---|
| Reactant Au/BSA | Conventional Gel-Exclusion[a] | Penefsky Gel-Exclusion[b] | Ultrafiltration[c] |
| 1.0 | 0.05 | 0.56 | 0.90 |
| 2.5 | 0.26 | ----- | 2.1 |
| 5.0 | ----- | 2.0 | 3.3 |

[a]Sephadex G-50 column (1 x 25 cm) eluted by gravity flow with 100 mM $NH_4HCO_3$ Buffer, pH 7.9; 1.1 mM albumin.
[b]Sephadex G-25 column (2 ml syringe) eluted centrifugally with 10 mM phosphate buffer, pH 7.4 + 100 mM NaCl; 0.4 mM albumin.
[c]Filtron® concentrators (3000 MW cutoff) centrifuged at 5000 x g for 45 min; 10 mM phosphate buffer, pH 7.4 + 100 mM NaCl; 0.4 mM albumin.

Table II shows the results of the measurements. When one equivalent of $Au(CN)_2^-$ was reacted with BSA, the Au/BSA ratio found after separation by conventional chromatography, Penefsky chromatography and ultrafiltration were 0.05, 0.56 and 0.90, respectively. Clearly the amount of protein gold binding observed is dependent on the separation technique employed. The ultrafiltration results confirm the NMR results: extensive association of $Au(CN)_2^-$ with albumin. The

NMR experiment is conducted in a single vessel without separation and it, like the ultrafiltration, maintains the equilibrium between free and bound aurocyanide. The Penefsy columns produce results that are intermediate between the equilibrium result and the elution over a large column by gravity flow.

Analysis of the ultrafiltration data establishes that there are two classes of equilibrium binding sites [29] and provides equilibrium constants for them:

$$Au(CN)_2^- + \text{Albumin} \underset{}{\overset{K_1 = 5.5\times10^4,\ n_1 = 0.8}{\rightleftharpoons}} \text{Albumin}\bullet(Au(CN)_2^-)_T$$

$$Au(CN)_2^- + \text{Albumin}\bullet(Au(CN)_2^-)_T \overset{K_2 = 5.5\times10^5,\ n_2 = 3}{\rightleftharpoons} \text{Albumin}\bullet(Au(CN)_2^-)_T(Au(CN)_2^-)_W$$

The labile equilibrium binding of aurocyanide to albumin provides a conceptual basis for rationalizing the disparate results of the three separation methods. At equilibrium, extensive binding is observed and, as expected for the association constants, the measured Au/BSA ratio is large. The conventional columns were eluted over a period exceeding an hour. The albumin moves through the column more rapidly than the trailing low molecular weight species, allowing the $Au(CN)_2^-$ to dissociate and be irreversibly lost. The Penefsky columns are rapidly eluted. During the short time that the albumin is in contact with the resin (< 1 min) there is relatively less opportunity for dissociation so that an intermediate value is obtained.

### DISCUSSION

The oxidation of gold(I) to gold(III) was found previously for AuSTm [4,5] and here for AuSTm, $Au(STm)_2^-$, AuSTg, $Au(CN)_2^-$ and auranofin to be complete with micromolar concentrations of gold and hypochlorite. The plausibility of these findings can be evaluated using standard reduction potentials [31,32]:

$$HOCl + H^+ + 2e^- = Cl^- + H_2O \qquad E_{o,½} - +1.49 \text{ volts}$$

$$AuCl_4^- + 2e^- = AuCl_2^- + 2Cl^- \qquad E_{o,½} = +0.93 \text{ volts}$$

We use here the potential for gold chlorides which is more meaningful than that for the hypothetical and non-existent aquo complexes, $Au^+$ and $Au^{+3}$. This approach is appropriate since the results of Fig. 2 require oxidation of the thiolate ligand in AuSTm to proceed gold oxidation and chloride is likely to ligate gold thereafter. For the net reaction, the potential is

$$AuCl_2^- + HOCl + H^+ + Cl^- = AuCl_4^- + H_2O \quad E_{o,rxn} = +0.56 \text{ volts}$$

Using the well known relationship $E_o$ = (RT/nF)log K, one can calculate an equilibrium constant and convert it to an apparent value, $K_{app}$, at physiological conditions (150 mM chloride ion and pH 7.4):

$$K_{app} = 10^{10.73}\ M^{-1} = [AuCl_4^-]/[AuCl_2^-][HOCl]$$

The ratio of Au(III) to Au(I) can be estimated as $K_{app}$ x [HOCl]. Assuming a minimum concentration of 1 μM HOCl is maintained during the oxidative burst, the numerical ratio exceeds 105. In other words, the conversion of gold(I) to gold(III) should be complete under conditions of the oxidative burst. (This result is independent of the selection of the acid or basic solution $E_o$ value for $HOCl/OCl^-$).

Graham *et al.* [23] have demonstrated that myleoperoxidase-generated hypochlorite can convert physiological concentrations of $SCN^-$ to $CN^-$ which in the presence of AuSTm reacts further to form $Au(CN)_2^-$. It has been shown here that $Au(CN)_2^-$ can be oxidized to Au(III). If an excess of cyanide is present, $Au(CN)_4^-$ will form due to the thermodynamically favorable and kinetically facile ligand exchange reactions that favor cyanide over chloride, thiocyanate or water ligands. Thus, the gold(III) species formed *in vivo* may be $Au(CN)_4^-$ as shown in the flow diagram of Fig 6. The UV spectrum of $Au(CN)_4^-$ is featureless in the region 200-500 $cm^-$ and the complex would not be detected by UV-vis spectroscopy. Further studies of this interesting possibility, using other detection methods, are planned.

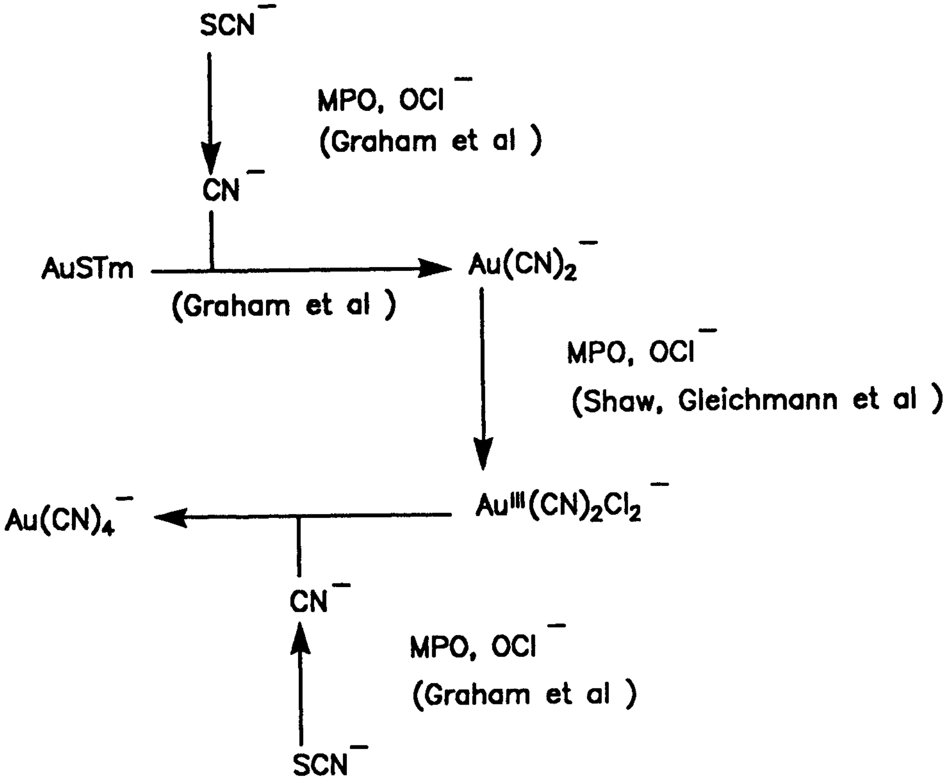

**Figure 6.** Is $Au(CN)_4^-$ a metabolite of gold(I) drugs? The scheme show a plausible route based on the work here and previous results of Rudkowski et al. [23] by which it may form during the oxidative burst.

The favorable thermodynamics and the rapidity of the oxidation of gold(I) by thiomalate suggest that a gold(I)-gold(III) redox cycle can be established in vivo:

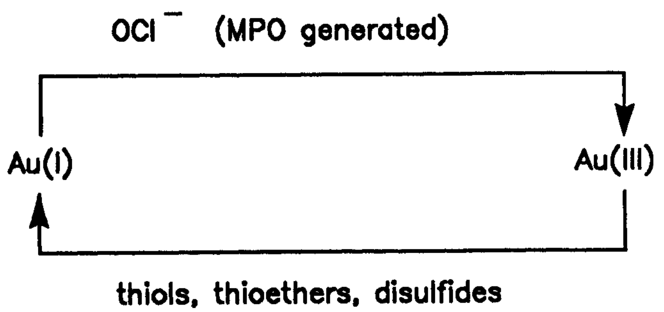

Our studies of the reduction of $AuCl_4^-$ and $Au(CN)_4^-$ by serum albumin and various thiols and thioethers (Isab, Jagaralmudi, Schraa and Shaw, unpublished) suggest that the reduction is somewhat slower than hypochlorite oxidation of gold(I). When the forward reaction occurs as rapidly as hypochlorite is generated, as found here, and the reverse reaction over a few minutes, kinetic considerations imply that a significant fraction of the gold present at the site of the oxidative burst can accumulate in the form of gold(III). This powerful oxidant can then diffuse away from the site and react with protein reductants that are encountered.

This, in turn, can have immunological consequences associated with both the clinical benefits and the side effects of chrysotherapy. Oxidation of proteins can convert self-proteins into "non-self proteins" causing peptides presented by HLA molecules of antigen-presenting cells to elicit immune responses as found in several side effects to gold therapy. Alternatively, this oxidation could alter the structure of proteins that are already being detected as non-self in the pathogenesis of rheumatoid arthritis. This could, in turn, inhibit their binding to HLA molecules, and, therefore, minimize or inhibit their subsequent recognition by T-cells leading to an observable clinical improvement in the disease state.

Further research on the details of gold(III) formation and its re-reduction by model proteins and the consequences of such reactions for the generation of cryptic peptides during antigen processing are crucial to a more detailed understanding of the mechanism of action of gold-based anti-arthritic agents.

### REFERENCES

1. Shaw, C. F. III *Comments Inorg. Chem.* **8**, 233-267 (1989).

2. Schuhmann, D.; Kubicka-Muranyi, M.; Mirtschewa, J.; Günther, J.; Kind, P.; Gleichmann, E. *J. Immunol.* **145**, 2132-2139 (1990).

3. Verwilghen, J.; Kingsley, G. H.; Gambling, L.; Panyai, G. S. *Arthr. Rheum.* **35**, 1413-1418 (1992).

4. Beverly, B. J.; Couri, D. *Fedn. Proc.* **46**, 3138 (1997).

5. Beverly, B. J. PhD Thesis, The Ohio State University, (1987).

6. Shaw, C. F. III; Witkiewicz, P. L. *JCS Chem. Commun.*, 1111-1114 (1981).

7. Shaw, C. F. III; Cancro, M. P.; Witkiewicz, P. L.; Eldridge, J. *Inorg. Chem.* **19**, 3198-3201 (1980).

8. Elder, R. C.; Eidness, M. K.; Heeg, M. J.; Tepperman, K. G.; Shaw, C. F. III *ACS Symposium Ser.* **209**, 285-400 (1983).

9. Sadler, P. J. *Struct. Bonding* **29**, 171-214 (1976).

10. Brown, D. H.; Smith, W. E. *Chem. Soc. Reviews* **9**, 217-239 (1980).

11. Shaw, C. F. III *Inorg. Persp. Med. Biol.* **2**, 278-355 (1979).

12. Smith, W. E.; Reglinski, J. *Persp. Bioinorg. Chem.* **1**, 183-208 (1991).

13. Shaw, C. F. III, in S. P. Fricker, Ed., *Metal Ions in Cancer Therapy,* Chapman and Hall Publishers, 1994, in press.

14. Brown, D. H.; McKinley, G. C.; Smith, W. E. *J. Chem. Soc., Dalton Trans.*, 199-201 (1978).

15. Grootveld, M. J.; Blake, D. R.; Sahinoclu, T.; Claxson, A. W. D.; Mapp, P.; Stevens, C.; Allen, R. E.; Furst, A. *Free Rad. Res. Commun.* **10**, 199-220 (1990).

16. Rudkowski, R.; Graham, G.; Champion, G. D.; Ziegler, J. B. *Biochem. Pharmacol.* **39**, 1687-1695 (1990).

17. Weiss, S. J.; Klein, R.; Slivka, A.; and Wei, M. *J. Clin. Invest.* **70**, 598-607, (1982).

18. Hormann, A. L., PhD Thesis, University of Wisconsin-Milwaukee, 1985.

19. Lewis, G.; Shaw, C. F. III *Inorg. Chem.* **25**, 58-62 (1986).

20. Graham, G. G.; Bales, J. R..; Grootveld, M. C.; and Sadler, P. J. *J. Inorg. Chem.* **25**, 163-173 (1985).

21. Isab, A. A.; Ghazi, I. H., Wazeer, I. M.; Perzanowski, H. P. *J. Inorg. Biochem.* **52**, 299-304 (1993).

22. (a) Dubinskii, V. I.; Shul'man, V. M.; Peshchevitskii, B. I. *Zh. Neorg. Khim.* **13**, 54 (1968). (b) Carlsson, L.; Lundgren, G. *Acta Chem. Scand.* **21**, 89 (1967). (c) Peshchevitskii, B. I.; Belevantsev, V. I.; and Kurbatova, N. V. *Zh. Neorg. Khim.* **16**, 1898 (1971)

23. Graham, G. G. and Dale, M. M. *Biochem. Pharmacol.* **39**, 1697-1702 (1990).

24. Elder, R. C.; Zhao, Z.; Zhang, Z.; Dorsey, J. G.; Hess, E. V.; Tepperman, K. J. *Rheumatol.* **20**, 268-272 (1993).

25. Small, H.; Stevens, T. S.; Baumann, W. O. *Anal. Chem.* **47**, 1801-1809 (1975).

26. Graham, G. G.; Haavisto, T. M.; Jones, H. M.; Champion, G. D. *Biochem. Pharmacol.*, **33**, 1257-1262 (1984).

27. Shaw, C. F. III; Isab, A. A.; Dunemann, L.; Gleichmann, E.; Turfeld, M.; Schraa, S. submitted for publication.

28. Penefsky, H. S. *J. Biol. Chem.* **252**, 2891-2899 (1977).

29. Brouwer, M.; Hoexum-Brouwer, T.; Cashan, R. E. *Biochemistry* **294**, 219-225 (1993).

30. Ford-Smith, M. H.; Habeeb, J. J.; Rawsthorne, J. H. *J.C.S. Dalton Trans.*, 2116-2120 (1972).

31. Pudephatt, R. J. *The Chemistry of Gold*, Elsevier Publishers, Amsterdam, 1978, pp. 18-21.

32. Hunsburger, J. F. in *CRC Handbook of Chemistry and Physics*, Chemical Rubber Co. Press, Boca Ratan, 1980, D155-D160.

